# Yield and Efficiency of Mental Health Screening: A Comparison of Screening Protocols at Intake to Prison

**DOI:** 10.1371/journal.pone.0154106

**Published:** 2016-05-11

**Authors:** Michael S. Martin, Beth K. Potter, Anne G. Crocker, George A. Wells, Ian Colman

**Affiliations:** 1 School of Epidemiology, Public Health and Preventive Medicine, University of Ottawa, Ottawa, Ontario, Canada; 2 Department of Psychiatry, McGill University and Douglas Mental Health University Institute Research Centre, Montreal, Quebec, Canada; University of Pennsylvania, UNITED STATES

## Abstract

**Background:**

The value of screening for mental illness has increasingly been questioned in low prevalence settings due to high false positive rates. However, since false positive rates are related to prevalence, screening may be more effective in higher prevalence settings, including correctional institutions. We compared the yield (i.e. newly detected cases) and efficiency (i.e. false positives) of five screening protocols to detect mental illness in prisons against the use of mental health history taking (the prior approach to detecting mental illness).

**Methods and Findings:**

We estimated the accuracy of the six approaches to detect an Axis I disorder among a sample of 467 newly admitted male inmates (83.1% participation rate). Mental health history taking identified only 41.0% (95% CI 32.1, 50.6) of all inmates with mental illness. Screening protocols identified between 61.9 and 85.7% of all cases, but referred between 2 and 3 additional individuals who did not have a mental illness for every additional case detected compared to the mental health history taking approach. In low prevalence settings (i.e. 10% or less) the screening protocols would have had between 4.6 and 16.2 false positives per true positive.

**Conclusions:**

While screening may not be practical in low prevalence settings, it may be beneficial in jails and prisons where the prevalence of mental illness is higher. Further consideration of the context in which screening is being implemented, and of the impacts of policies and clinical practices on the benefits and harms of screening is needed to determine the effectiveness of screening in these settings.

## Introduction

Between a quarter and half of individuals with severe mental illness receive appropriate treatment, both in the general population [1,2] and in institutional settings such as jails and prisons [3,4]. While screening is an intuitive solution to improve uptake of services, it is resource intensive. As jail and prison inmates have higher rates of mental disorder [5] that are often undetected [3,4], screening for mental illness is commonly recommended [6]. While there are a number of studies examining the psychometric properties of screening tools in jails and prisons, there is no evidence examining the conditions under which screening improves outcomes compared to prior case detection practices [7]. The costs and benefits of screening must be carefully weighed to choose which screening test(s)—if any—will work best in the specific context [8,9]. Increasing the detection of cases of mental illness (i.e. increasing sensitivity or screening yield) is typically the primary focus of screening, given that delays in treatment are associated with a worse prognosis [10,11]. However, false positives (i.e. low specificity and positive predictive values) can overburden resources [12,13]. They may also have risks such as stigma for the false positive patient [14]. If false-positive screening results are not identified by clinical staff providing follow-up, in addition to the costs of providing treatment, there may also be the risk of adverse outcomes such as medication side-effects [15] and abuse [16] for the individual. Effective triage following screening is thus an important component to reducing costs of unnecessary treatments and any potential consequences of being falsely identified by screening [17].

There is no clear guidance on the levels of accuracy that define an acceptable screening tool [7]. There is increasing recognition that screening may not be effective in the general population due to low positive predictive values and evidence that new cases detected by screening are often of mild severity that do not benefit from treatment [18,19]. No single screening tool has been shown to detect more than approximately 70–75% of illness among prisoners, and low specificity is an issue [7,20]. Multiple tests can be used to increase sensitivity (i.e. by referring anyone exceeding the cut-offs on either test, which we refer to as simple cut-offs) or to increase specificity (i.e. by requiring the cut-offs on multiple tests to be exceeded). This approach of combining multiple tests has been taken in Canadian and New Zealand prisons [7,17,21], although the added value of multiple versus a single test is unclear at this time.

Because sensitivity and specificity are generally constant properties of a test, they are most commonly reported. They indicate the percentage of persons with an illness who screen positive (sensitivity) and the percentage of persons without illness who screen negative (specificity). However, sensitivity and specificity work backwards from the outcome to the screening result, which is the opposite of how clinicians use screening in practice. The positive and negative predictive values conversely start from the screening result, and indicate the percentage of individuals referred by screening who are in fact ill (positive predictive value) and the percentage of individuals who fall below the cut-off scores who are not ill (negative predictive value). This information is useful to clinicians, who (ideally in consultation with the patient) must judge whether the likelihood of illness is sufficiently high to initiate treatment or to pursue further testing [22].

While they are more clinically useful, positive and negative predictive values vary in relation to the prevalence of illness [23,24]. In relative terms, a positive screening result is typically associated with a constant increase in the probability that a person has illness (if test accuracy varies in different sub-groups, in particular those that are related to illness severity, these estimates may be biased and thus vary when applied in practice [25]). In absolute terms the probability a person who is sampled from a higher prevalence group (i.e. a prison) is more likely to be ill than a person from a lower prevalence group (i.e. the general population). Since screening does not change a person's baseline risk, the positive predictive value of a test (a measure of the probability that a person with a positive screen is ill) will be higher when applied in the higher prevalence setting [24].

The current study compared the screening yield (i.e. rate of newly identified cases of illness) and efficiency (i.e. rate of false positives) of various screening protocols to detect mental illness in prisons as compared to the prior detection method.

## Methods

This study was conducted following the STAR-D guidelines (see S1 Table for the completed checklist). All procedures were approved by the Ottawa Health Science Network Research Ethics Board (protocol number 20150240-01H). As we undertook secondary analysis of data collected in the course of routine screening of inmates, and from a research study conducted by Correctional Service of Canada (CSC) to estimate the prevalence of mental illness in prison [26], informed consent for our specific project was not obtained. CSC obtained written consent from inmates at two points–prior to completing mental health screening and prior to participating in the prevalence study–which included a statement that de-identified data may be used for research purposes consistent with the Privacy Act [27]. De-identified data were provided by CSC in four separate data files, which we combined by matching on the random study ID code assigned by the CSC analyst: (1) demographic variables (i.e. sex, age, race) and results of the gold standard diagnostic interview; (2) mental health screening results; (3) admissions to treatment centres (accredited hospitals) for intensive mental health treatment and (4) mental health services provided by mental health professionals in regular prisons (i.e. primary care).

### Sample

**Fig 1:**
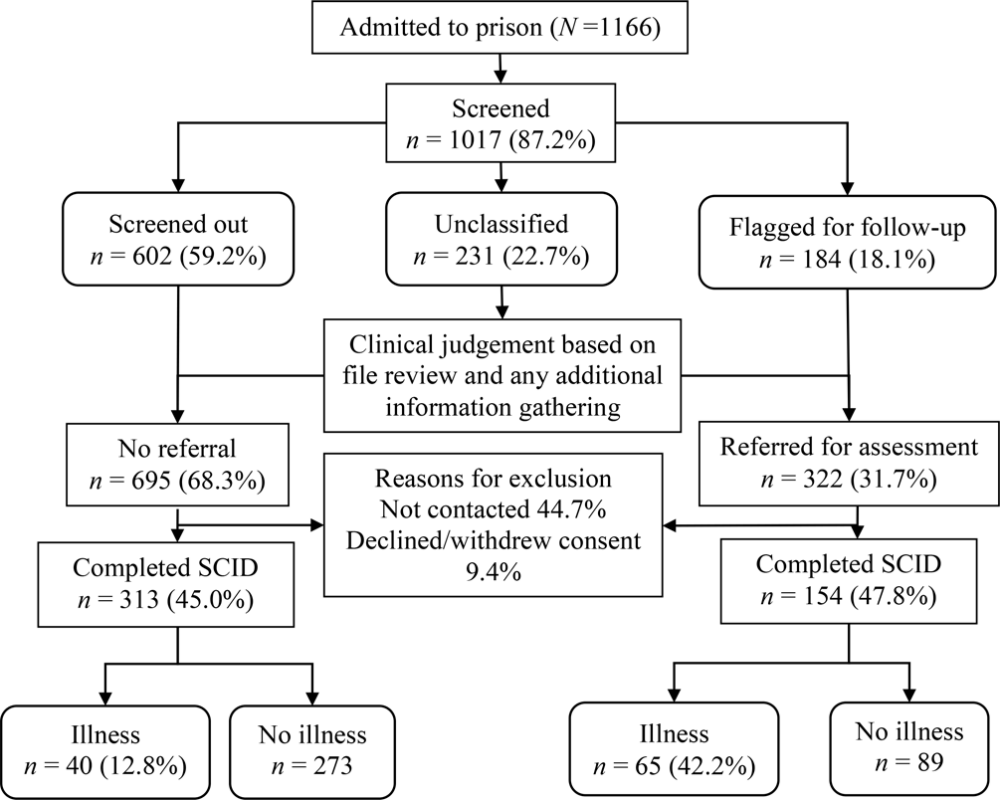
Screening process and participant flow diagram.

Participants in the current study were those who participated in screening (as part of routine practice) and the diagnostic interview (for research purposes to estimate the prevalence of illness in CSC prisons). The final sample consisted of 467 male inmates admitted to prisons in the provinces of Manitoba, Saskatchewan, and Alberta between January and June 2013, and in the province of Quebec between January and September 2014. Because there were different sampling frames for screening and the prevalence study, we evaluated potential selection biases by defining our eligible study population as all inmates who completed screening between the earliest and latest dates on which inmates who participated in the clinical interviews completed screening (N = 1,017). Of these eligible inmates, 562 (55.3%) were invited to participate in the prevalence study, of whom 83.1% (*n* = 467) agreed to participate. To ensure that there was no verification bias [28], we compared the 467 inmates included in our sample to the 550 inmates who refused or withdrew their consent prior to completing the interview (*n* = 95; 9.4%) or were not approached to participate (*n* = 455; 44.7%). The participation rates were similar for inmates who were referred for follow-up services following screening (47.8% of screened individuals completed the gold standard) and those who were not (45.0% of screened individuals completed the gold standard; Fig 1). Participants and non-participants were also similar in terms of age (mean age of 36 for both groups) and ethnicity. Among participants, 61% self-reported white race, 24% identified as Aboriginal, and 14% reported belonging to other minority ethnic groups. Among those without a structured diagnostic interview these proportions were similar: 63%, 22%, and 14% respectively.

### Measures and procedure

#### Screening

Inmates complete the computerized screening within 14 days of admission. The screening includes four standardized mental health screening tools—the Brief Symptom Inventory (BSI) [29], the Depression Hopelessness Suicide Screening Form (DHS) [30], the General Ability Measure for Adults [31] and the Adult Self-Report Screening Scale for Attention-Deficit Hyperactivity Disorder [32] (because the latter two tools screen for intellectual functioning and ADHD, which are not the focus of this study, we do not do not discuss them further). Screening also includes nine mental health history indicators. Three of these indicators pertain to the inmate’s current status (diagnosis, psychotropic medication use, hospitalization prior to incarceration). Prior to the implementation of screening, these three indicators were used identify mental illness and to monitor the prevalence of mental health needs among inmates [33]. Thus, endorsement of any of these three indicators provides a baseline method of case detection at intake to prison against which screening protocols could be compared. The remaining six indicators concern lifetime mental health diagnoses, treatments and self-harm.

The BSI includes 53 items, to which the respondent indicates the frequency at which they have experienced various symptoms of distress in the past 7 days on a scale from 0 (never) to 4 (always). Three overall distress scores and nine subscale scores are calculated by taking the average of the items relevant to that scale. The nine subscales are somatisation, obsessive-compulsive, interpersonal-sensitivity, depression, anxiety, hostility, phobic anxiety, paranoid ideation, and psychoticism. Three overall distress scores reflect the overall rate of distress (the Global Severity Index), the number of symptoms endorsed (the Positive Symptom Total) and the intensity of endorsed symptoms (the Positive Symptom Distress Index). The test authors recommend that a T-score of 63 (based on general population norms) or higher on the Global Severity Index or any 2 of the 9 sub-scales should be used to define 'caseness' (i.e. likely mental illness) [29].

The scale comprises 39 true-false items, which produce subscale scores for depression, hopelessness and a total score. The DHS includes ten “critical items” that inquire about current suicide ideation, thoughts supportive of suicide, and historical suicide indicators. Two additional critical items inquire about a past diagnosis of depression and whether the inmate knows someone who has completed suicide. However, slightly more than half of all inmates endorsed one of these twelve items, and few offered incremental predictive validity in the prediction of incidents of self-injury or suicide attempts during the first 180 days following intake to prison [34]. Using a subset of five items reflecting more recent or frequent histories of self-harm and current suicide ideation, the referral rate decreased to 17.7%, with a sensitivity of 84.2% and a specificity of 82.6%. Previously recommended cut-off scores for the DHS are a depression scale score of 7 or higher, a hopelessness score of 2, a total score of 8 or higher [35,36] or any of the 5 critical items regarding current or recent suicide ideation or attempts [34].

Initially, CSC implemented screening where an inmate would be referred if they exceeded a T-score of 65 on the Global Severity Index or any 2 of the 9 subscales on the BSI, if they exceeded a T-score of 60 on any of the DHS scales, or if they reported any of the 12 critical items for suicide risk on the DHS. A preliminary validation study using un-blinded clinical judgment found that this model had a referral rate of 62%, a sensitivity of 86% and a specificity of 52% [37]. In order to reduce the false positive rate, CSC implemented a tree-based scoring model that was developed using the Iterative Classification Tree (ICT) approach to incorporate the multiple tests and mental health history indicators. The model uses recursive partitioning techniques to identify combinations of scores on the various screening tests that best discriminate individuals with mental illness from those without. Groups with a high probability of mental illness (i.e. who score high on multiple scales) are classified as flagged (i.e. referred for further assessment or treatment), and groups with a low probability of illness (i.e. low scores on multiple scales) are classified as screened out. Inmates who have ambiguous screening results (i.e. score high on some scales but low on others), are designated as unclassified. For inmates who are designated as unclassified, clinicians have discretion whether to refer the inmate (at a minimum they are required to review information from the inmate’s medical and prison files). In order to determine staff decisions for unclassified inmates, we retrieved service use data in the 90 days following screening from CSC’s electronic records of mental health service contacts and transfers to Treatment Centres. The model was estimated to have a sensitivity between 56 and 88% and a specificity between 69 and 95% depending on how well clinicians responded to unclassified inmates [37]. However, this performance has yet to be replicated in an independent sample.

#### Gold-standard diagnostic interview

Inmates were interviewed by a research assistant to complete the mood, anxiety and psychotic disorder modules of the Structured Clinical Interview for DSM-IV [38] and the modified Global Assessment of Functioning (GAF) Scale [39] as part of the mental health prevalence study conducted by CSC’s research branch. Given that by definition mental illness should cause moderate to severe symptoms or impairment [40], the case definition for this study was a current diagnosis of a mood, psychotic or anxiety disorder plus a GAF score of 60 or less [39]. Diagnostic categories were not mutually exclusive, and thus an inmate could be diagnosed with multiple disorders. Interviewers were blind to screening results, and diagnostic interview results were not shared with screening staff. Interviews typically occurred after screening (*n* = 431; 92.3%), with a range from 38 days prior to screening to 83 days after screening. As only nine (1.9%) participants received treatment between completing screening and the diagnostic interview, it is unlikely that any bias introduced by treatment between the two tests would materially change our findings.

### Analysis

We sought to validate five decision rules that were previously developed and that were embedded within the current battery of screening tests in Canadian prisons. Specifically we validated (1) the ICT model (the decision rule that is currently used when reviewing screening results); (2) the BSI alone (a T-score of 63 or greater on the Global Severity Index, or on two of the nine sub-scales) [29]; (3) the DHS alone (a depression scale score of 7 or higher, a hopelessness score of 2, a total score of 8 or higher [35,36] or any of the 5 critical items regarding current or recent suicide ideation or attempts [34]); (4) referral for an inmate who exceeds the cut-offs on either of the BSI or DHS (which we refer to as simple cut-offs) (5) referral for an inmate who exceeds the cut-offs on both the BSI and DHS (referred to as multiple cut-offs). We compared these screening protocols to the prior case detection method used in Canadian prisons of gathering mental health history information and referring an inmate reporting a current diagnosis, medication use, or recent hospitalization.

We calculated the sensitivity, specificity, and positive and negative predictive values (PPV and NPV) and 95% confidence intervals for each case detection method. The sensitivity of each method to detect mood, psychotic and anxiety disorders are also reported separately as past research suggests higher detection of psychotic than mood disorders [3,4,17].We also report standardized true positive, true negative, false negative and false positive rates per 1,000 inmates screened. Because these rates are most relevant clinically (i.e. they reflect the impact of screening on clinical caseloads) we discuss the results primarily in these terms. These rates are used to identify the conditions under which screening would provide a net benefit. In the absence of exact costs and benefits of screening, the ratio of how many additional false positives would be identified by screening to identify each additional case can be used to compare options and determine the conditions under which screening would provide a net benefit [41]. If for example there were ten false positives for every true positive, the benefits of treating each true positive would have to be at least ten times greater than the costs associated with false positives to offset the fact that in absolute numbers false positives are more common.

As sensitivity analyses, we calculated the expected number of false positives per additional case detected if the screening protocols were implemented in settings with different prevalence rates and with a lower prior detection rate as drawn from a prior study in British prisons [4]. Since sensitivity and specificity are generally independent of prevalence [23], we conducted the sensitivity analyses in three steps: (1) calculate the number of cases and non-cases based on the prevalence; (2) for each case detection method, estimate the number of true positives and false negatives based on the sensitivity and the number of true negatives and false positives using the specificity; (3) calculate the ratio of false positives per true positive for each screening approach compared to the alternative case detection method (these steps are illustrated in S1 File).

## Results

In total, 105 participants (22.5%) met the case definition for mental illness. Table 1 presents the performance of the various protocols to accurately classify inmates’ mental health status. 16.3% of inmates were referred based on history taking, whereas the various screening protocols had referral rates between 33.0% and 56.7%. Under the history taking approach, of every 1,000 screenings, only 92 individuals with mental illness are referred, whereas 133 individuals with mental illness are not referred (sensitivity: 41.0%, 95% CI 32.1, 50.6). Under the various screening protocols, between 139 and 193 individuals with mental illness are referred for every 1,000 screenings (sensitivity ranges from 61.9% to 85.7%). All screening protocols increased the detection rate of psychosis (sensitivity ranges from 84.2 to 94.7%) by approximately one third compared to history taking (sensitivity of 63.2%). However, because of the lower prevalence, there was minimal difference in absolute numbers of detected cases of psychotic disorders (i.e. approximately 2–4 per 1,000 inmates screened) between the screening protocols. The more sensitive screening protocols (e.g. the BSI or DHS alone, or the use of simple cut-offs) result primarily in higher numbers of detected mood and anxiety disorders compared to the more specific screening protocols (e.g. the ICT or the use of multiple cut-offs on the BSI and DHS). For example, of 45 additional true positives per 1000 inmates screened using the simple cut-offs there were an additional 26 detected mood disorders and 34 anxiety disorders (recall that inmates could be diagnosed with multiple disorders). For each additional illness detected by any of the screening protocols, between 2 and 3 additional individuals without illness are also referred relative to history taking. For the screening protocols to be beneficial compared to the prior approach, the benefits of treating a true positive must be at least double the harms associated with a false positive result.

**Table 1:** Accuracy (95% CI) of 6 approaches to detect mental illness.

	History taking	ICT	Multiple cut-offs	BSI	DHS	Simple cut-offs
Referral rate	16.3 (13.2, 19.9)	33.0 (28.9, 37.4)	33.2 (29.1, 37.6)	44.3 (39.9, 48.8)	45.6 (41.1, 50.1)	56.7 (52.2, 61.1)
True positives/1000 screens	92	139	148	171	169	193
Mood disorder	54	81	96	109	107	122
Anxiety disorder	60	92	101	118	116	135
Psychotic disorder	26	34	34	39	36	39
False positives/1000 screens	71	191	184	272	287	375
Extra false positives per true positive compared to history taking	—	2.6	2.0	2.5	2.8	3.0
False negatives/1000 screens	133	86	77	54	56	32
Mood disorder	75	47	32	19	21	6
Anxiety disorder	103	71	62	45	47	28
Psychotic disorder	15	6	6	2	4	2
True negatives/1000 screens	704	585	591	503	488	400
Sensitivity	41.0 (32.1, 50.6)	61.9 (52.3, 70.6)	65.7 (56.2, 74.1)	76.2 (67.2, 83.3)	75.2 (66.1, 82.5)	85.7 (77.7, 91.1)
Mood disorder	41.7 (30.1, 54.3)	63.3 (50.6, 74.4)	75.0 (62.8, 84.2)	85.0 (73.9, 91.9)	83.3 (71.9, 90.7)	95.0 (86.3, 98.3)
Anxiety disorder	36.8 (26.8, 48.0)	56.6 (45.4, 67.2)	61.8 (50.6, 71.9)	72.4 (61.5, 81.2)	71.1 (60.1, 80.1)	82.9 (72.9, 89.7)
Psychotic disorder	63.2 (41.1, 80.9)	84.2 (62.4, 94.5)	84.2 (62.4, 94.5)	94.7 (75.3, 99.1)	89.5 (68.6, 97.1)	94.7 (75.3, 99.1)
Specificity	90.9 (87.5, 93.4)	75.4 (70.7, 79.6)	76.2 (71.6, 80.3)	64.9 (59.9, 69.6)	63.0 (57.9, 67.8)	51.7 (46.6, 56.8)
PPV	56.6 (45.4, 67.2)	42.2 (34.7, 50.1)	44.5 (36.9, 52.4)	38.6 (32.2, 45.4)	37.1 (30.9, 43.8)	34.0 (28.6, 39.9)
NPV	84.1 (80.1, 87.4)	87.2 (83.0, 90.5)	88.5 (84.5, 91.6)	90.4 (86.2, 93.4)	89.8 (85.5, 92.9)	92.6 (88.1, 95.5)

As noted previously, these ratios depend on the prevalence. Table 2 presents the sensitivity analyses, where we calculated the absolute number of false positives per additional true positive for each screening protocol (holding sensitivity and specificity constant) in settings with different prevalence rates. If the prevalence is 10% or less, the screening protocols would result in 4.6 to 16.2 false positives for every additional detected case compared to mental health history taking. Conversely, in settings with a prevalence of 40% the consequences of false negatives must only be similar to those of false positives for screening to be beneficial. In settings where prior detection rates are lower (e.g. in UK prisons only 25% of inmates with mental illness and 3% without mental illness were assessed by in-reach teams [42]), these ratios are slightly less, but the same pattern emerges that screening is less effective in low prevalence settings.

**Table 2:** Number of extra false positives per true positive for varying levels of prevalence and prior detection rates.

Prevalence	ICT	Multiple cut-offs	BSI	DHS	Simple cut-offs
Compared to history taking (41% sensitivity; 90.9% specificity)					
5%	13.4	10.7	13.7	14.7	16.2
10%	6.7	5.3	6.7	7.4	7.8
15%	4.1	3.3	4.1	4.6	4.9
20%	3.0	2.3	2.9	3.3	3.5
25%	2.2	1.8	2.2	2.4	2.6
30%	1.7	1.4	1.7	1.9	2.1
35%	1.4	1.1	1.4	1.5	1.6
40%	1.1	0.9	1.1	1.2	1.3
Compared to detection from Senior et al (2012; 25% sensitivity and 97% specificity)					
5%	10.8	9.4	11.7	12.5	13.9
10%	5.3	4.6	5.6	6.1	6.7
15%	3.3	2.9	3.5	3.8	4.2
20%	2.3	2.0	2.5	2.7	3.0
25%	1.8	1.5	1.9	2.0	2.2
30%	1.4	1.2	1.5	1.6	1.7
35%	1.1	0.9	1.2	1.3	1.4
40%	0.9	0.8	0.9	1.0	1.1

## Discussion

**Fig 2:**
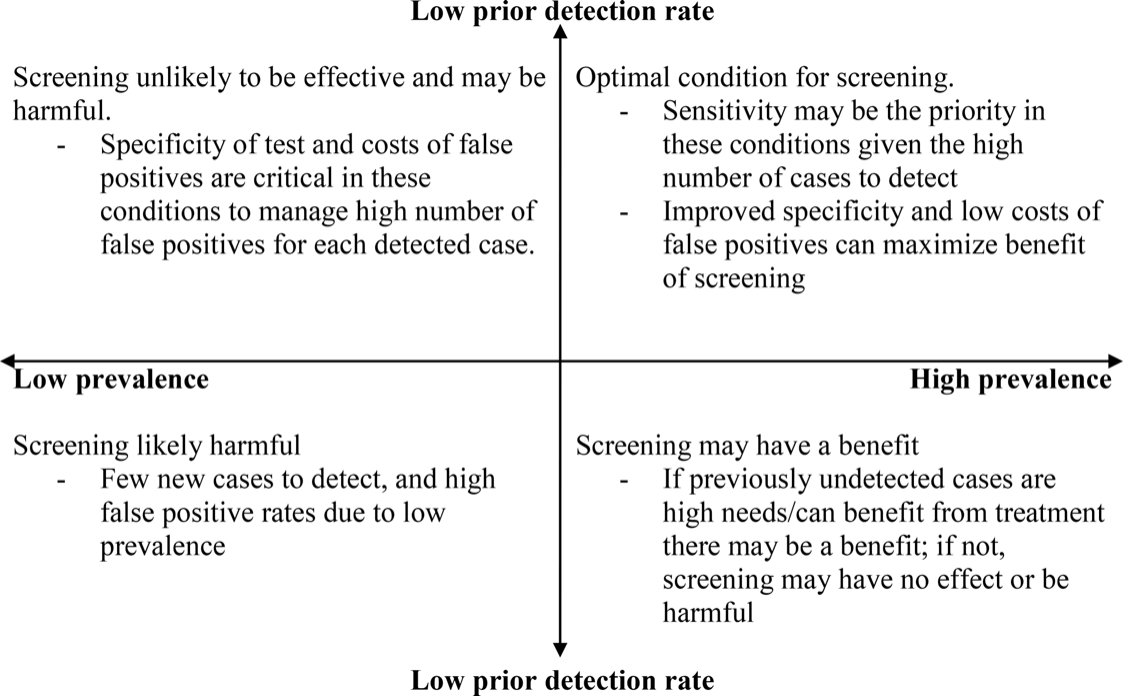
Relationship between prevalence, prior detection rate and potential impact of screening.

Studies on mental health screening typically do not evaluate the yield of new cases and efficiency of screening relative to usual clinical detection, which may over-estimate both the accuracy and value of screening [7,25]. The ratio of how many additional false positives screening generates in order to detect each new case helps illustrate how tools of varying levels of sensitivity and specificity perform in practice depending on the prevalence of mental illness and the prior levels of detection of mental illness. Others have proposed that this ratio can be used to inform decision making about whether the benefits of screening (e.g. preventing events associated with illness and/or improving recovery rates) outweigh the harms (e.g. costs, inconvenience, and harms of treatments that are inappropriately provided to those who are not ill), after taking into account the relative importance of both types of errors [41]. It is noteworthy, that in higher prevalence settings, the number of additional false positives per newly detected case is similar for each of the five screening protocols as compared to history taking. This suggests that despite the emphasis on the psychometric properties of screening tools in the literature, the effectiveness of screening likely depends much more on characteristics of the screened population, and system-level practices and policies that are associated with benefits (e.g. treatments that improve outcomes) for newly detected cases and minimize the costs (e.g. effective triage to avoid un-necessary treatments) for false positives. Since these population characteristics, and practices and policies will likely vary across settings, it is not possible to make unequivocal recommendations about screening. Therefore, in Fig 2, we summarize our findings regarding conditions under which screening is more likely to be beneficial and when it may be harmful.

Our findings are consistent with prior research that screening is inefficient in settings with low prevalence, even if detection rates of illness are low (e.g. community settings) [18,43,44]. This perspective is reflected by the rates of roughly 5 to 16 false positives per newly detected case in our sensitivity analyses for a prevalence of either 5% or 10%. Screening is more efficient when the prevalence of illness is high, and in particular when prior detection is low (the upper right quadrant of Fig 2). Under these conditions, there will be the greatest number of new cases to detect through screening, and the proportion of false positives will be lower [24]. Nonetheless, the ratio of 2 to 3 false positives per additional true positive indicate that after accounting for cases that would be detected in the absence of screening only one quarter [i.e. 1/(3+1)] to one third [i.e. 1/(1+2)] of new referrals will be for people with a mental illness.

The effectiveness of screening depends on provision of appropriate follow-up of inmates with elevated scores. While this question has received little attention, recent studies in the United Kingdom [45] and Australia [46] both found that approximately 25% of inmates identified by screening did not receive follow-up. Conversely, a recent study in New Zealand showed that mental health caseloads had doubled within 2 years of implementing screening (from approximately 5 to 10%) despite a low screening rate of only 25%. Further work is needed to examine the effect of screening generated referrals on longer-term outcomes. A meta-analysis found that counseling interventions in primary care were more effective for individuals with depression identified through routine clinical practice versus those identified by screening [47]. Inmates with psychotic disorders and mental health histories are often detected by staff even without screening [3,4], suggesting that these might be the highest need cases based on obvious signs of impairment. Symptoms may resolve naturally for up to half of all inmates reporting depression and anxiety at intake [48,49]. Therefore, many individuals with mental illness that is detected only through screening require little more than close monitoring. It may be of particular value to determine whether individuals who are detected only through longer and more sensitive screening benefit from treatment to the same extent as those who are identify by shorter, more specific screening. If they do not, developing referral pathways that prioritize the urgency of follow-up (see for example the PolQuest [50] screening tool) may be an effective strategy.

The prior discussion has focused on patient outcomes, which should be the primary consideration when deciding whether to screen. Nonetheless, screening results contribute valuable information that can be used for research, quality improvement and resource allocation decisions. Routine screening may be a cost-effective and timely way of monitoring changes in rates of mental health symptoms over time, between institutions, or between groups of inmates, and provides valuable information for examining outcomes of persons with mental illness. Accurate estimates of psychometric properties of screening tools can be used in sensitivity/bias analyses in such studies [51]. From an organizational perspective, if the costs of excess assessments are less than the costs that would be devoted to other quality improvement and research activities, screening would be a value added activity that could support better patient outcomes.

## Conclusions

Our findings suggest that screening may be beneficial in higher prevalence settings such as jails and prisons. However, given the lack of empirical evidence about the harms and benefits it is unclear how much benefit screening may provide or whether this is cost-effective. It is important to consider the circumstances unique to the specific context prior to implementing screening (e.g. the prevalence of illness and the current detection rates) to identify whether the conditions are likely to be favourable for the implementation (or continuation) of mental health screening. Given the lack of data about the impact of screening, the yield and efficiency of screening compared to existing practices can provide some insight into the potential value of screening. If screening is implemented or further evaluated through randomized controlled trials to establish its effectiveness, policies and practices that minimize costs and maximize benefits of screening should be considered to increase the likelihood that screening will lead to improved outcomes.

## Supporting Information

S1 File Illustration of sensitivity analyses. (DOCX)

S1 Table STAR-D checklist. (DOC)
