# Supplementary material for: Yield and Efficiency of Mental Health Screening: A Comparison of Screening Protocols at Intake to Prison
Source: PLoS One. 2016 May 11;11(5):e0154106. doi: 10.1371/journal.pone.0154106 (PMC4864401; doi:10.1371/journal.pone.0154106)
Supplement: S1 File — (DOCX) [file pone.0154106.s001.docx]

Calculations for sensitivity analyses.

Step 1 : Calculate number of true positives, false positives, true negatives and false negatives per 1,000 inmates for the comparison approach to detecting mental illness (mental health history taking)

For example, for a prevalence of 5% (sensitivity of mental health history taking of 41.0% and specificity of 90.9%)

|  | No illness | Illness | Total |
| --- | --- | --- | --- |
| Screened out | 863 (950 x 90.9%) | 30 (50 – 20) | 893 (863 + 30) |
| Screened in (referred) | 87 (950-863) | 20 (50 x 41.0%) | 107 (87 + 20) |
| Total | 950 (1000-50) | 50 (1000 x 5%) | 1000 |

Step 2: Calculate number of true positives, false positives, true negatives and false negatives per 1,000 screenings based on the prevalence of mental illness, sensitivity and specificity of the screening.

For example, for a 5% prevalence, for the ICT model (sensitivity of 61.9% and specificity of 75.4%).

|  | No illness | Illness | Total |
| --- | --- | --- | --- |
| Screened out | 716 (950 x 75.4%) | 19 (50 – 31) | 735 (716 + 19) |
| Screened in (referred) | 234 (950-716) | 31 (50 x 61.9%) | 265 (234 + 31) |
| Total | 950 (1000-50) | 50 (1000 x 5%) | 1000 |

Step 3: Calculate the number of extra false positives per additional detected case (true positive)

$\frac{(False positives for screening-False positives for history taking}{True positives for screening-True positives for history taking}$ = $\frac{(234-87)}{(31-20)}$ = $\frac{147}{11}$ = 13.4

Repeat steps 2 and 3 for each screening protocol and prevalence value.

To compare against a different case detection method, repeat step 1 for the new comparison method, and repeat steps 2 and 3 for each screening protocol and prevalence value as compared to this new comparison standard.
