# Supplementary material for: Yield and Efficiency of Mental Health Screening: A Comparison of Screening Protocols at Intake to Prison
Source: PLoS One. 2016 May 11;11(5):e0154106. doi: 10.1371/journal.pone.0154106 (PMC4864401; doi:10.1371/journal.pone.0154106)
Supplement: S1 Table — (DOC) [file pone.0154106.s002.doc]

# STARD checklist for reporting of studies of diagnostic accuracy

*(version January 2003)*

| **Section and Topic** | **Item**  **#** |  | **On page #** |
| --- | --- | --- | --- |
| TITLE/ABSTRACT/  KEYWORDS | 1 | Identify the article as a study of diagnostic accuracy (recommend MeSH heading 'sensitivity and specificity'). | Keyword sensitivity and specifity used  Abstract indicates that “We compared the accuracy of five screening protocols to detect mental illness in prisons against the use of mental health history taking (the prior approach to detecting mental illness) to identify scenarios under which different screening approaches best balance benefits and harms. |
| INTRODUCTION | 2 | State the research questions or study aims, such as estimating diagnostic accuracy or comparing accuracy between tests or across participant groups. | Page 2: “The current study used screening data from a sample of Canadian prisoners to (1) estimate the accuracy of various previously developed screening protocols to detect mental illness in prison; and (2) compare the conditions under which the benefits of screening would outweigh its harms.” |
| METHODS |  |  |  |
| *Participants* | 3 | The study population: The inclusion and exclusion criteria, setting and locations where data were collected. | Page 2 (see Figure 1 for STAR-D flow diagram)  “This study involved secondary analysis of data for a sample of 467 male inmates collected in the course of routine screening of inmates, and from a research study conducted by Correctional Service of Canada (CSC) to estimate the prevalence of mental illness in prison. Data were collected between January and June 2013 in the provinces of Manitoba, Saskatchewan, and Alberta, and between January and September 2014 in the province of Quebec. Inmates were eligible for the current study if they completed screening during this time (N = 1,017) and if they completed the gold standard psychiatric interview (described below). As the prevalence study used a different sampling frame from the current study, only 562 (55.3%) inmates who completed screening were invited to participate in the prevalence study, of whom 83.1% (n = 467) agreed to participate.” |
|  | 4 | Participant recruitment: Was recruitment based on presenting symptoms, results from previous tests, or the fact that the participants had received the index tests or the reference standard? | Page 2 (see paragraph for inclusion/exclusion criteria explaining that recruitment is based on completion of the index test and reference standard) |
|  | 5 | Participant sampling: Was the study population a consecutive series of participants defined by the selection criteria in item 3 and 4? If not, specify how participants were further selected. | Yes – consecutive series |
|  | 6 | Data collection: Was data collection planned before the index test and reference standard were performed (prospective study) or after (retrospective study)? | Retrospective study (see page 2).  This study involved secondary analysis of data for a sample of 467 male inmates collected in the course of routine screening of inmates, and from a research study conducted by Correctional Service of Canada (CSC) to estimate the prevalence of mental illness in prison. |
| *Test methods* | 7 | The reference standard and its rationale. | Page 4:  “Inmates were interviewed by a research assistant to complete the mood, anxiety and psychotic disorder modules of the Structured Clinical Interview for DSM-IV(29) and the modified Global Assessment of Functioning (GAF) Scale(30) as part of the mental health prevalence study. Interviewers were blind to screening results, and diagnostic interview results were not shared with prison staff. Interviews typically occurred after screening (*n* =431; 92.3%), with a range from 38 days prior to screening to 83 days after screening. Given that by definition mental illness should cause moderate to severe symptoms or impairment(31), the case definition for this study was a current diagnosis of a mood, psychotic or anxiety disorder plus a GAF score of 60 or less(30).” |
|  | 8 | Technical specifications of material and methods involved including how and when measurements were taken, and/or cite references for index tests and reference standard. | See pages 3-4 for details about timing of administration of screening and reference standard and references for the tests |
|  | 9 | Definition of and rationale for the units, cut-offs and/or categories of the results of the index tests and the reference standard. | Cut-offs were based on prior research described on page 5:  “we validated (1) the ICT model (the decision rule that is currently used when reviewing screening results); (2) the BSI alone (a T-score of 63 or greater on the Global Severity Index, or on two of the nine sub-scales)(23); (3) the DHS alone (a depression scale score of 7 or higher, a hopelessness score of 2 or higher(32) or any of the 5 critical items regarding current or recent suicide ideation or attempts(33)); (4) referral for an inmate who exceeds the cut-offs on either of the BSI or DHS (which we refer to as simple cut-offs) (5) referral for an inmate who exceeds the cut-offs on both the BSI and DHS (referred to as multiple cut-offs). We compared these screening protocols to the prior case detection method used in Canadian prisons of gathering mental health history information and referring an inmate reporting a current diagnosis, medication use, or recent hospitalization.“ |
|  | 10 | The number, training and expertise of the persons executing and reading the index tests and the reference standard. | Index tests were completed and interpreted using computers (see page 3). |
|  | 11 | Whether or not the readers of the index tests and reference standard were blind (masked) to the results of the other test and describe any other clinical information available to the readers. | Double blinding was ensured; the readers of the index and reference tests never had access to the results of the other test.  Page 4: “Interviewers were blind to screening results, and diagnostic interview results were not shared with prison staff.” |
| *Statistical methods* | 12 | Methods for calculating or comparing measures of diagnostic accuracy, and the statistical methods used to quantify uncertainty (e.g. 95% confidence intervals). | Page 5: We calculated the sensitivity, specificity, and positive and negative predictive values (PPV and NPV) and 95% confidence intervals for each case detection method. The sensitivity of each method to detect mood, psychotic and anxiety disorders are also reported separately as past research suggests higher detection of psychotic than mood disorders(3,4,14). We also report standardized true positive, true negative, false negative and false positive rates per 1,000 inmates screened. |
|  | 13 | Methods for calculating test reproducibility, if done. | Not done as this was a replication of previously developed tests. |
| RESULTS |  |  |  |
| *Participants* | 14 | When study was performed, including beginning and end dates of recruitment. | Page 4: “Data were collected between January and June 2013 in the provinces of Manitoba, Saskatchewan, and Alberta, and between January and September 2014 in the province of Quebec.” |
|  | 15 | Clinical and demographic characteristics of the study population (at least information on age, gender, spectrum of presenting symptoms). | Pages 2-3. “The participation rates were similar for inmates who were referred for follow-up services following screening (47.8% of screened individuals completed the gold standard) and those who were not (45.0% of screened individuals completed the gold standard; see Figure 1). Participants and non-participants were also similar in terms of age (mean age of 36 for both groups) and ethnicity. Among participants, 61% self-reported white race, 24% identified as Aboriginal, and 14% reported belonging to other minority ethnic groups. Among those without a structured diagnostic interview these proportions were similar: 63%, 22%, and 14% respectively.”  Prevalence of any psychotic, mood or anxiety disorder with moderate to severe impairment was 22.5% (see page 6). The prevalence by disorder can be calculated from Table 1 by adding true positives and false negatives and dividing by 1000. This gives the prevalence of 4% for psychotic disorder, 12.8% for mood disorder and 16.3% anxiety disorder |
|  | 16 | The number of participants satisfying the criteria for inclusion who did or did not undergo the index tests and/or the reference standard; describe why participants failed to undergo either test (a flow diagram is strongly recommended). | See figure 1 and pages 2-3: “The participation rates were similar for inmates who were referred for follow-up services following screening (47.8% of screened individuals completed the gold standard) and those who were not (45.0% of screened individuals completed the gold standard; see Figure 1). Participants and non-participants were also similar in terms of age (mean age of 36 for both groups) and ethnicity. Among participants, 61% self-reported white race, 24% identified as Aboriginal, and 14% reported belonging to other minority ethnic groups. Among those without a structured diagnostic interview these proportions were similar: 63%, 22%, and 14% respectively.” |
| *Test results* | 17 | Time-interval between the index tests and the reference standard, and any treatment administered in between. | Page 4: “Interviews typically occurred after screening (n =431; 92.3%), with a range from 38 days prior to screening to 83 days after screening. Nine (1.9%) participants received treatment between completing screening and the diagnostic interview, suggesting that it is unlikely that treatment substantial bias our estimates of the performance of screening.” |
|  | 18 | Distribution of severity of disease (define criteria) in those with the target condition; other diagnoses in participants without the target condition. | Page 4: “Given that by definition mental illness should cause moderate to severe symptoms or impairment(31), the case definition for this study was a current diagnosis of a mood, psychotic or anxiety disorder plus a GAF score of 60 or less(30).” |
|  | 19 | A cross tabulation of the results of the index tests (including indeterminate and missing results) by the results of the reference standard; for continuous results, the distribution of the test results by the results of the reference standard. | Table 1: The values of the cross-tabulation are provided in 4 rows (true positives, true negatives, false positives and false negatives); these numbers have been standardized as rates per 1,000 tests to increase generalizability and ease of presentation, and can be converted back to unstandardized values by dividing by 2.14 (1000/467) if required. |
|  | 20 | Any adverse events from performing the index tests or the reference standard. | As this is secondary analysis we have no access to this information, but do not anticipate there having been any adverse events. |
| *Estimates* | 21 | Estimates of diagnostic accuracy and measures of statistical uncertainty (e.g. 95% confidence intervals). | See table 1 for sensitivity, specificity, PPV and NPV with 95% confidence intervals |
|  | 22 | How indeterminate results, missing data and outliers of the index tests were handled. | Indeterminate results exist only for the model currently in use by the prison service. As noted on page 4: “For inmates who are designated as unclassified, clinicians have discretion whether to refer the inmate (at a minimum they are required to review information from the inmate’s medical and prison files). In order to determine staff decisions for unclassified inmates, we retrieved service use data in the 90 days following screening from CSC’s electronic records of mental health service contacts and transfers to Treatment Centres.” |
|  | 23 | Estimates of variability of diagnostic accuracy between subgroups of participants, readers or centers, if done. | Not done |
|  | 24 | Estimates of test reproducibility, if done. | Not done |
| DISCUSSION | 25 | Discuss the clinical applicability of the study findings. | See discussion section on pages 9-11 |
